# Random Forest Model in the Diagnosis of Dementia Patients with Normal Mini-Mental State Examination Scores

**DOI:** 10.3390/jpm12010037

**Published:** 2022-01-04

**Authors:** Jie Wang, Zhuo Wang, Ning Liu, Caiyan Liu, Chenhui Mao, Liling Dong, Jie Li, Xinying Huang, Dan Lei, Shanshan Chu, Jianyong Wang, Jing Gao

**Affiliations:** 1 Department of Neurology, State Key Laboratory of Complex Severe and Rare Diseases, Peking Union Medical College Hospital, Chinese Academy of Medical Science and Peking Union Medical College, Beijing 100730, China; wangjie_smu@163.com (J.W.); liucy-pumch@163.com (C.L.); maochenhui@pumch.cn (C.M.); sophie_d@163.com (L.D.); jielicathy@126.com (J.L.); hxypumch@163.com (X.H.); ld94616@163.com (D.L.); chuss9486@163.com (S.C.); 2 Department of Computer Science and Technology, Tsinghua University, Beijing 100084, China; wang-z18@mails.tsinghua.edu.cn (Z.W.); victorliucs@gmail.com (N.L.)

**Keywords:** machine learning, dementia, cognitive dysfunction, neuropsychological tests, mental status and dementia tests

## Abstract

**Background:** Mini-Mental State Examination (MMSE) is the most widely used tool in cognitive screening. Some individuals with normal MMSE scores have extensive cognitive impairment. Systematic neuropsychological assessment should be performed in these patients. This study aimed to optimize the systematic neuropsychological test battery (NTB) by machine learning and develop new classification models for distinguishing mild cognitive impairment (MCI) and dementia among individuals with MMSE ≥ 26. **Methods:** 375 participants with MMSE ≥ 26 were assigned a diagnosis of cognitively unimpaired (CU) (*n* = 67), MCI (*n* = 174), or dementia (*n* = 134). We compared the performance of five machine learning algorithms, including logistic regression, decision tree, SVM, XGBoost, and random forest (RF), in identifying MCI and dementia. **Results:** RF performed best in identifying MCI and dementia. Six neuropsychological subtests with high-importance features were selected to form a simplified NTB, and the test time was cut in half. The AUC of the RF model was 0.89 for distinguishing MCI from CU, and 0.84 for distinguishing dementia from nondementia. **Conclusions:** This simplified cognitive assessment model can be useful for the diagnosis of MCI and dementia in patients with normal MMSE. It not only optimizes the content of cognitive evaluation, but also improves diagnosis and reduces missed diagnosis.

## 1. Introduction

The prevalence of dementia is rising with the aging of the population, affecting the quality of life and increasing the burden on society and the family [1]. Mild cognitive impairment (MCI) is considered a transitional stage between normal aging and dementia, with a higher risk of developing dementia. The diagnosis of MCI and dementia early has prognostic value [2,3].

The most widely used screening tool for dementia is the Mini-Mental State Examination (MMSE) [4], a 30-point instrument that assesses several domains including orientation, attention, language, memory, and executive function. MMSE has good sensitivity and specificity for detecting dementia. Creavin et al. reported that in the community, a pooled sensitivity of 0.85 and specificity of 0.90 at a cut point of 24, and sensitivity of 0.87 and specificity of 0.82 at a cut point of 25 [5]. Pooled estimates of 15 studies showed a sensitivity of 0.89 and specificity of 0.89 at a cut point of 23 or less or 24 or less [6]. However, the sensitivity (0.20–0.93) and specificity (0.48–0.93) to detect MCI vary significantly in different studies, meaning less consistent estimates for test accuracy [6]. Thus, its ability to distinguish between cognitively impaired subjects and cognitively unimpaired (CU) adults is limited [7,8,9], leading to the possibility that some patients with normal MMSE scores but cognitive impairment may be missed.

For these individuals with normal MMSE scores, a more comprehensive cognitive assessment is needed. The systematic neuropsychological test battery (NTB) designed by the Peking Union Medical College Hospital (PUMCH) consists of more than 20 subtests to evaluate five cognitive domains: executive function, visuospatial ability, language, memory, and abstract reasoning and calculation [10]. It takes into account Chinese culture and language and is suitable for the Chinese elderly to detect MCI and dementia. All these subtests have been used and validated in the Chinese population, and normative population data were available. However, administering such a comprehensive battery is time-consuming.

Recent studies had shown that machine learning (ML) exhibited excellent performance in identifying MCI and dementia [11,12,13,14,15,16,17], but these mostly used biomarker data such as neuroimaging and CSF components that were expensive technologies [12,13,16]. ML diagnostic models based on cognitive data were gradually being applied [11,15,18,19]. Random forest (RF), an ensemble ML method based on a set of decision trees, has positive significance in processing complex neuropsychological data and excellent predictive performance for the diagnosis of cognitive impairment [15]. Using the feature selection method in RF, we can determine the importance of features and delete insignificant ones, thereby reducing the complexity of the NTB.

Therefore, the purpose of this study was to use RF to simplify the NTB and shorten evaluation time. Several important neuropsychological subtests were selected, and new RF models were developed to classify CU, MCI, and dementia for people with normal MMSE scores.

## 2. Materials and Methods

### 2.1. Participants

375 (67 CU adults, 174 MCI patients and 134 dementia patients) participants were enrolled consecutively from the PUMCH dementia cohort, the Dementia Clinic of the Department of Neurology of PUMCH between May 2009 to April 2021. They received a detailed clinical evaluation that included medical history taking, physical and neurological examinations, a systemic of neuropsychological tests, laboratory testing, and neuroimaging studies (head CT or MRI). The inclusion criteria included MMSE score ≥ 26, with normal function in motor, sensory, balance, reflex, and ability to complete all neuropsychological tests. Patients with significant functional disabilities, a history of major psychiatric illness, or any other central nervous system disorders other than cognitive impairment were excluded. 

### 2.2. Neuropsychological Examinations

Cognitive tests included the Chinese version of the MMSE [20] and the PUMCH version of Montreal cognitive assessment (MoCA-P) [10]. Previous studies had shown that MMSE scores were influenced by age, gender, and particularly years of education [9]. Several studies that investigated the normative data of the MMSE in the Chinese population got different optimal cut-off points ranging from 19 to 26 for dementia screening [9,21,22]. In this study, we defined ≥26 points as normal MMSE scores. A Chinese version of ADL was used to determine impairment in everyday functioning [23], which was revised and supplemented according to the scale of Lawton and Brody [24], consisting of eight activities focused on instrumental ADL (IADL) (including using telephone, shopping, food preparation, housekeeping, laundry, transportation, managing medications, and handling finances) and 12 activities focused on the basic ADL (BADL) (e.g., dressing, bathing, eating, getting in or out of bed, using the toilet and so on). Each item of ADL range from 1 to 4 (1 = can do it myself, 2 = have some difficulty doing but can still do it by myself, 3 = need help to do it, 4 = cannot do it at all). The lowest ADL score was 20 points, indicating that the patient’s ability was completely normal, and the highest was 80 points. The Hospital Anxiety and Depression (HAD) scale was used to screen for anxiety and depression among patients [25]. Participants were administered the above assessments as the diagnostic neuropsychological measures.

All subjects underwent the systemic NTB to evaluate five cognitive domains. These were: (1) Executive function: category verbal fluency [26], the digit symbol test (DST) [27], the trail making test A (TMT A) [28], the clock drawing test [8], paired-associate learning (PAL) of The Clinical Memory Test [29], the block design test of the Aphasia Battery of Chinese [30], and modified Luria three-step task [31]; (2) Visuospatial ability: the block design test and figure copying of the Aphasia Battery of Chinese [30], the copy of a modified Rey-Osterrieth figure [32], and gestures imitation; (3) Language: several subtests of the Aphasia Battery of Chinese including spontaneous speech, auditory comprehension, repetition, and naming [30]; (4) Memory: PAL, the logical memory test (LMT) of the modified Wechsler Memory Scale [33], and the auditory verbal learning test-Huashan version (AVLT-H) [34] were used to assess verbal memory. Nonverbal memory was measured by the modified Rey-Osterreith with a 10-min free recall; and (5) Abstract reasoning and calculation: subtests of the Wechsler Adult Intelligence Scale including similarities and calculations [27]. All subtests of NTB were not used to assist in making the clinical diagnosis of MCI or dementia, but as screening tests for machine learning.

### 2.3. Diagnostic Criteria

A clinical diagnosis of CU, MCI, or dementia was made based on all available information including clinical history and neuropsychological measures. MCI and dementia were diagnosed based on clinical judgment and/or on cognitive test performance according to the clinical criteria of the National Institute on Aging and the Alzheimer’s Association (NIA-AA) guidelines [35,36,37]. Dementia diagnostic criteria included the following: evidence of decline from a previous level of cognitive performance; cognitive impairment diagnosed through history-taking and/or cognitive assessment; evidence of impairment in activities in daily living (ADL score > 23, IADL score > 11). MCI diagnostic criteria included the following: evidence of decline from a previous level of cognitive performance; no evidence of impairment in activities in daily living (ADL score ≤ 23, IADL score ≤ 11); not meeting the criteria for dementia. Subjects in the CU group had no or only mild cognitive decline, and neuropsychological tests were in the normal range.

### 2.4. Statistical Analysis

Continuous variables were described as mean ± standard deviation (M ± SD) and categorical variables as numbers and percentages (*n*, %). ANOVA with Bonferroni post-hoc tests or chi-square analysis was applied to detect significant differences between the different subgroups. A *p*-value of <0.05 was considered statistically significant. Statistical analysis was performed by SPSS version 24.0 software (Chicago, IL, USA).

### 2.5. Machine Learning

We manually extracted 64 features, including basic demographic information (sex, age, education years, etc.) and neuropsychological scores of NTB. All features were listed in Supplementary Table S1. At first, we used RF to calculate the importance of all features and perform feature selection. We tested all features with five-fold cross-validation and used mean area under the curves (AUC) as the performance metric. Different features had different importance in diagnosing dementia. Selecting the top-ranked features and filtering out the bottom-ranked features can simplify the classification process.

Next, other classification models, including logistic regression, decision tree, SVM, and XGBoost were trained and compared with RF. The performance of various models was evaluated by accuracy, precision, recall, F1 score, and AUC.

After selecting the features with high importance or the features we were interested in, 5-fold cross-validation was employed to train classification models, and the corresponding receiver operating characteristic (ROC) curves were also plotted. For each model, we got three ROC curves to distinguish CU, MCI, and dementia. The performance of each model effectiveness was evaluated using the mean ROC of the 5-fold cross-validation, the mean AUC, sensitivity, and specificity. AUC takes a value between 0 and 1, where AUC = 1 represents perfect diagnostic accuracy. Sensitivity is the true positive rate and specificity is the true negative rate. Sensitivity and specificity were calculated according to the maximal Youden’s Index (sensitivity + specificity−1).

Classification models were built by using Python 3.7.9 with the package scikit-learn 0.23.2.

## 3. Results

### 3.1. Participants’ Characteristics

375 participants, 161 men and 214 women, aged 65.51 ± 11.46 years, were recruited. Of these, 67 (17.9%) were CU, 174 (46.4%) had MCI, and 134 (35.7%) had dementia. Table 1 shows the baseline demographic and cognitive profiles of the three groups. The dementia group was significantly older than the MCI group, and years of education were significantly higher in the CUs than in the subjects with MCI and dementia. There was no significant gender difference between the three groups. For MMSE and MoCA-P scores, CU > MCI > dementia (*p* < 0.001); for ADL, IADL and BADL, CU = MCI < dementia.

### 3.2. Assessment of Feature Importance

**Figure 1:**
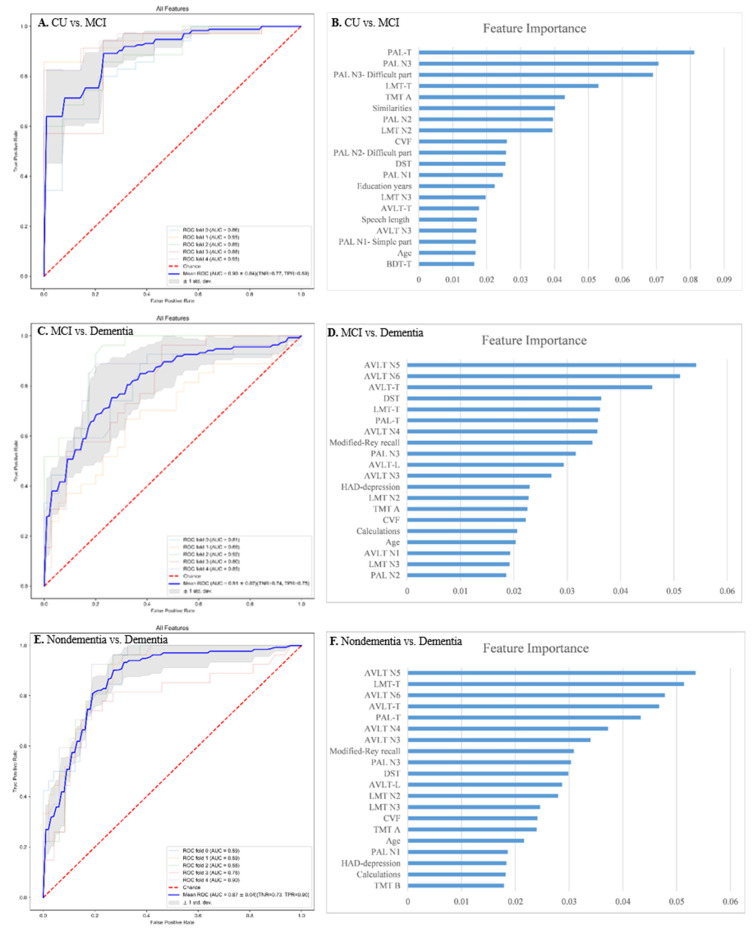
Receiver operating characteristic (ROC) curve analysis for the detection of MCI and dementia and the optimal 20 features. (**A**) ROC curve of all features for the detection of MCI from CU. (**B**) 20 top-ranked features for the detection of MCI from CU. (**C**) ROC curve of all features for the detection of dementia from MCI. (**D**) 20 top-ranked features for the detection of dementia from MCI. (**E**) ROC curve of all features for the detection of dementia from non-dementia. (**F**) 20 top-ranked features for the detection of dementia from non-dementia. Abbreviations: AVLT N1 = the first learning trial of AVLT-H (auditory verbal learning test-Huashan version); AVLT N3 = the third learning trial of AVLT-H; AVLT N4 = the fourth short delayed free recall trial of AVLT-H; AVLT N5 = the fifth long delayed free recall trial of AVLT-H; AVLT N6 = the sixth delayed category cue recall trial of AVLT-H; AVLT-L = total score of AVLT N1, N2,and N3; AVLT-T = total score of AVLT N1, N2, N3, N4 and N5; BDT-T = total score of the block design test; CVF = category verbal fluency; DST = Digit Symbol Test; HAD = hospital anxiety and depression; LMT N2 = the second story of logical memory test (LMT); LMT N3 = the third story of LMT; LMT-T = total score of LMT; PAL N1 = The first learning trial of PAL (paired-associate learning); PAL N1-Simple part = simple word pairs of PAL N1; PAL N2 = The second learning trial of PAL; PAL N2-Difficult part = difficult word pairs of PAL N2; PAL N3 = The third learning trial of PAL; PAL N3-Difficult part = difficult word pairs of PAL N3; PAL-T = total score of PAL N1, N2, and N3; TMT A = trail making test A; TMT B = trail making test B.

We extracted all features (64 features) into the RF classification model and calculated feature importance. ROC analysis for the detection of MCI and dementia and the top 20 features were shown in Figure 1. ROC-AUC of all features for distinguishing MCI from CU was 0.90 ± 0.04, sensitivity and specificity were 0.89 and 0.77 (Figure 1A), and the most important feature was PAL-T (total score of the three learning trials of PAL) (Figure 1B). ROC-AUC of all features for distinguishing dementia from MCI was 0.81 ± 0.07, sensitivity and specificity were 0.75 and 0.74 (Figure 1C), and the most important feature was AVLT N5 (the fifth long-delayed free recall trial of AVLT-H) (Figure 1D). ROC-AUC of all features for distinguishing dementia from non-dementia was 0.87 ± 0.04, sensitivity and specificity were 0.90 and 0.73 (Figure 1E), and the most important feature was AVLT N5 (Figure 1F).

### 3.3. Performance of Various Classification Models

Table 2 shows the performance of various classification models. The accuracies of the logistic regression, decision tree, SVM, XGBoost, and RF models were 0.605, 0.597, 0.624, 0.664, and 0.680, while the AUCs were 0.796, 0.696, 0.809, 0.816, and 0.852. Among these methods, The RF classifier achieved the most stable performance with high accuracy compared with other classifiers.

### 3.4. Selecting the Optimal Neuropsychological Tests to Establish Diagnostic Models

Finally, we selected six interested neuropsychological subtests with 22 high importance features (including AVLT-H, PAL, modified Rey figure, LMT, DST, and TMT A). The selected features contained in each neuropsychological subtest were listed in Supplementary Table S2. These features trained four new RF diagnosis models. The Performance (ROC AUC, sensitivity, and specificity) of these four models were shown in Table 3. If we selected three selected subtests (AVLT-H, PAL, and modified Rey figure) with 19 features to establish the diagnosis model, AUC to detect CU from MCI, MCI from dementia, dementia from nondementia was 0.86, 0.77, 0.84, respectively. If we selected four subtests (AVLT-H, PAL, modified Rey figure, and LMT) with 20 features, AUC to discriminate CU from MCI, MCI from dementia, dementia from non-dementia was 0.87, 0.79, 0.83. If we selected five subtests (AVLT-H, PAL, modified Rey figure, LMT, and DST) with 21 features, AUC to detect CU from MCI, MCI from dementia, dementia from nondementia was 0.86, 0.77, 0.84, respectively. When we chose all six important subtests with 22 selected features to establish the RF classification model, AUC to detect CU from MCI was 0.89 (sensitivity = 0.87 and specificity = 0.85), AUC to detect MCI from dementia was 0.79 (sensitivity = 0.84 and specificity = 0.63), and AUC to detect dementia from nondementia was 0.84 (sensitivity = 0.72 and specificity = 0.81). RF Model based on 22 neuropsychological features was almost equivalent to the model established using all 64 features. At the same time, the cognitive tests time was reduced from more than an hour to 30 min.

## 4. Discussion

The present study found that 35.7 percent of subjects with MMSE scores ≥ 26 had evidence of dementia. Similar results have been obtained from previous studies [38,39]. This suggests that MMSE, as the only cognitive testing tool, is not sufficient to diagnose cognitive impairment. According to the 2011 NIA-AA criteria of “dementia”, when clinical history and bedside cognitive tests cannot provide evidence of cognitive impairment, neuropsychological tests should be performed [36]. In this study, we applied the RF algorithm to determine the contribution of different cognitive tests and to screen out efficient neuropsychological features for better diagnosis of cognitive impairment. Our results showed that the RF algorithm has satisfactory performance in the task of diagnosing MCI (AUC = 0.89) and dementia (AUC = 0.84). The ML method helped develop a simplified version of NTB for CU, MCI, and dementia classification in patients with MMSE scores ≥ 26. The diagnostic model finally included six neuropsychological tests with highly important features, and other low-importance tests were deleted, thus greatly shortening the evaluation time.

The NTB is suitable for the Chinese cultural background and language habits, but the normative data of its subtests have not been updated for a long time. As the education level and living conditions of the Chinese have improved significantly in recent decades, the clinical value of the norms has been limited. Reestablishing the norms for large samples is time-consuming and requires organization and resources to conduct. In addition, the norms are influenced by many factors such as age, gender, education level, and residence (rural or urban). ML has the potential to solve the above problems by allowing multi-dimensional interactions between variables [15]. It also can rank variables that are critical to assessing cognitive impairment, which can be used to optimize neuropsychological testing [40,41]. RF can handle both linear and non-linear data and offers an advanced method to deal with outliers or missing values [42]. It has been used to solve classification and regression problems and can serve as a powerful tool to distinguish MCI and dementia [43]. Studies have found that the RF algorithm has excellent efficiency in diagnosing dementia based on neuropsychological testing [15]. Kleiman et al. reported that RF two-class classification showed greater clinical utility compared to the three-class approach in classifying cognitive impairment [44]. Therefore, our two-class models for distinguishing MCI from CU, dementia from MCI, or dementia from nondementia.

One review [45] that included 59 studies indicated that MMSE, as a global cognitive screening tool, showed the highest discrimination coefficient in the ML automatic classification of cognitive impairment. However, previous studies did not focus on people with normal MMSE scores when developing diagnostic models or optimizing neuropsychological tests using ML methods [45]. In these studies, subjects with MCI and mild dementia had significantly lower baseline scores on the bedside cognitive tests than our sample [11,41,44,46,47]. For example, Quintana et al. [47] reported that the mean MMSE score of the MCI group and dementia group was 25.77 ± 2.22, 20.37 ± 3.98, respectively. In the Chiu et al. [11] study, the mean MMSE and MoCA scores in the very mild dementia group were 19.7 ± 4.7, 12.4 ± 6.0, respectively. Lower MMSE scores indicate more severe impairment of cognition, and the diagnostic accuracy of the ML model developed based on this situation will be higher, which means that it is more difficult to detect dementia in people with normal MMSE. Classification models using ML on demographical and neuropsychological data in the literature showed wide heterogeneity in performance metrics. Weakley et al. [48] reported a sensitivity and specificity of 0.84 and 0.89 for differentiating MCI from CU, and 0.95 and 0.97 for dementia and CU, and Battista et al. [41] with 0.98 and 0.81 for MCI, and 1.00 and 0.96 for dementia. In this work, the selected sample were subjects whose MMSE was higher than the cut-off value. This is the first time to address the question that classifies people with normal MMSE. Our results showed that the RF model has good sensitivity (0.87) and specificity (0.85) for differentiating MCI from CU, as well as good sensitivity (0.85) and specificity (0.73) for dementia from nondementia.

RF had also been proven to be more effective in feature selection. Previous studies that focused on ML and cognitive measures had the disadvantage of having fewer neuropsychological features [47,49], or they just focused on the comparison between MCI and CU or CU and dementia [50,51]. Our study included 20 neuropsychological tests and compared CU, MCI, and dementia groups. The most frequent optimal neuropsychological tests reported in the literature were episodic memory [41,47,49] (like AVLT, logical memory test) and semantic fluency [46,47,52]. However, these neuropsychological measures mainly focus on Alzheimer’s disease and dementia and cannot examine the damage of multiple cognitive domains. In our research, the combination of six tests is sufficient to cover multiple cognitive domains including executive function, visual perception function, language, memory, and attention, which can help diagnose all-cause dementia. AVLT-H and LMT, which assess both immediate and delayed recall, are popular methods for detecting episodic memory impairment [53,54]. PAL measures the strength of memory binding of twelve word-pairs [29]. The word pairs are presented verbally, one pair at a time. Then the participant hears the first word of each word-pair and is asked to answer the last word. PAL assesses episodic memory and executive function and could successfully detect MCI and dementia [55,56]. Modified Rey includes copy and delayed recall of the complex figure, assessing visuospatial ability and nonverbal memory. Good performance of DST and TMT A requires intact motor speed, attention, and visual perception functions, which is an important executive domain involved in semantic information processing [57]. The 2011 NIA-AA staging criteria also suggests some neuropsychological tests that are considered to be predictors of conversion from MCI to dementia [33]. These tests are generally consistent with those selected in our study.

In addition, the RF algorithm could be used not only to optimize the NTB but also to simplify individual subtests. For example, AVLT-H begins with three learning trials, followed by the fourth short delayed free recall trial, the fifth long-delayed free recall trial, the sixth category cue recall trial, and the recognition trial [53]. When ranking variables’ importance, we found that AVLT N5 was the most important feature. Therefore, we choose to administer the first five trials of AVLT-H in the future practical application and delete the sixth category cue recall trial and the recognition trial. The second story of LMT was the best predictor among the three stories, so only the second story needs to be completed when performing this neuropsychological test.

There were two main limitations to this study. First, this study was a retrospective, single-center, observational study with inherent selection bias. Prospective, multi-centered, large-scale studies are therefore warranted. A second limitation is that we did not sub-classify dementia. Subjects in the dementia group were patients with all-cause dementia, most of which is Alzheimer’s disease and vascular dementia, and other dementia subtypes such as frontotemporal dementia and dementia with Lewy body were rare. This might cause some features to become less important. For example, language-related features such as repetition and naming were removed. Future research needs to consider dementia subtypes.

## 5. Conclusions

The present study showed that the RF algorithm can be a useful tool to classify CU, MCI, and dementia among a population with normal MMSE. We found that the optimized NTB, consisting of six neuropsychological tests (AVLT-H, PAL, modified Rey figure, LMT, DST, and TMT A), enables detection of MCI and dementia with good sensitivity and specificity. As cognitive markers, neuropsychological assessments have the excellent performance to identify cognitive disorders. For low- and middle-income countries, this has advantages over using classifiers based on more invasive, expensive, and time-consuming methods such as cerebrospinal fluid markers.

## Figures and Tables

**Table 1:** Comparison of demographic details and cognitive data among the groups.

	Total *n* = 375	CU *n* = 67	MCI *n* = 174	Dementia *n* = 134	χ^2^/F ^a^	Post Hoc Tests ^b,c^
Age (years)	65.51 ± 11.46	63.24 ± 12.00	64.16 ± 11.61	68.41 ± 10.44	7.05 **	1 = 2 < 3
Gender (% female)	214 (57.1%)	43 (64.2%)	99 (56.9%)	72 (53.7%)	1.99	-
Education years	12.28 ± 3.91	13.88 ± 3.34	11.93 ± 3.98	11.96 ± 3.92	6.63 **	1 > 2 = 3
MMSE	27.80 ± 1.31	28.70 ± 1.17	27.95 ± 1.22	27.15 ± 1.17	40.42 **	1 > 2 > 3
MoCA-P	24.35 ± 3.08	27.18 ± 1.65	24.64 ± 2.77	22.54 ± 2.82	71.52 **	1 > 2 > 3
ADL	24.34 ± 4.57	21.78 ± 2.05	22.26 ± 2.53	28.31 ± 4.85	136.32 **	1 = 2 < 3
IADL	11.39 ± 3.30	9.45 ± 1.82	9.82 ± 1.99	14.39 ± 3.11	160.18 **	1 = 2 < 3
BADL	12.95 ± 1.92	12.33 ± 0.73	12.45 ± 1.01	13.93 ± 2.69	31.29 **	1 = 2 < 3
HAD-anxiety	4.66 ± 3.38	4.45 ± 3.15	4.48 ± 3.52	5.01 ± 3.29	1.06	-
HAD-depression	4.88 ± 3.48	4.50 ± 3.50	4.46 ± 3.44	5.64 ± 3.41	4.86 *	1 = 2 < 3

Data were shown as mean ± standard deviation (SD) or frequency (percentage, %). ^a^ Test statistic: F = one-way ANOVA value; χ^2^ = chi-square test value. ^b^ 1: CU group; 2: MCI group; and 3: Dementia group. ^c^ Pairwise comparisons among the three groups of subjects were conducted using the Bonferroni post hoc tests. * *p* < 0.05; ** *p* < 0.001. Abbreviations: ADL = Activities of Daily Living; BADL = Basic ADL; CU = Cognitively Unimpaired; HAD = Hospital Anxiety and Depression; IADL = Instrumental ADL; MCI = Mild Cognitive Impairment; MMSE = Mini-Mental State Examination; MoCA-P = PUMCH version of Montreal Cognitive Assessment; PUMCH = Peking Union Medical College Hospital.

**Table 2:** Performance of models trained by various methods.

	Accuracy	Precision	Recall	F1 Score	ROC-AUC
Logistic Regression	60.53	60.80	60.08	60.12	79.62
Decision Tree	59.73	60.48	60.86	60.21	69.55
SVM	62.40	65.37	59.29	61.17	80.87
XGBoost	66.40	67.78	66.15	66.70	81.61
Random Forest	68.00	71.09	66.73	68.02	85.17

**Table 3:** Performance of the four new RF diagnosis models on the classification of CU, MCI, and Dementia.

New Diagnosis Models	Subtests of Interest	Number of Features	ROC AUC for CU vs. MCI (Sensitivity, Specificity)	ROC AUC for MCI vs. Dementia (Sensitivity, Specificity)	ROC AUC for Dementia vs. Nondementia (Sensitivity, Specificity)
Model-1	PAL, AVLT-H, Modified-Rey	19	0.86 (0.79, 0.84)	0.77 (0.68, 0.76)	0.84 (0.72, 0.81)
Model-2	PAL, AVLT-H, Modified-Rey, LMT	20	0.87 (0.78, 0.84)	0.79 (0.76, 0.66)	0.83 (0.70, 0.83)
Model-3	PAL, AVLT-H, Modified-Rey, LMT, DST	21	0.87 (0.83, 0.84)	0.79 (0.81, 0.65)	0.84 (0.84, 0.71)
Model-4	PAL, AVLT-H, Modified-Rey, LMT, DST, TMT A	22	0.89 (0.92, 0.74)	0.79 (0.84, 0.63)	0.84 (0.85, 0.73)

Abbreviations: AVLT-H = Auditory Verbal Learning Test-Huashan version; CU = Cognitively Unimpaired; DST = Digit Symbol Test; LMT = Logical Memory Test; MCI = Mild Cognitive Impairment; Modified-Rey = Modified Rey-Osterreith figure; PAL = Paired-Associate Learning.

## Data Availability

The data that support the findings of this study are available from the corresponding author upon reasonable request.

## Supplementary Materials

The following are available online at https://www.mdpi.com/article/10.3390/jpm12010037/s1, Table S1: 64 features that extracted, Table S2: Six neuropsychological subtests and they selected features.
