# Supplementary material for: Random Forest Model in the Diagnosis of Dementia Patients with Normal Mini-Mental State Examination Scores
Source: J Pers Med. 2022 Jan 4;12(1):37. doi: 10.3390/jpm12010037 (PMC8780625; doi:10.3390/jpm12010037)
Supplement: Supplementary file 1 [file jpm-12-00037-s001.zip › jpm-1500047-supplementary.pdf]

Supplementary material

Supplementary Table S1. 64 features that extracted.

Number	Features	Explanation notes
1	Age	years
2	Age group	< 65 years old; 65-85 years old; > 85 years old
3	Gender	Male or female
4	Education	years
5	Education group	≤6 years of education; >6 and ≤12 years of education; >12 years of education
6	Handedness	Right-handedness; left-handedness; Mixed- handedness
7	HAD-anxiety	Anxiety score of Hospital Anxiety and Depression scale
8	HAD-depression	Depression score of Hospital Anxiety and Depression scale
9	CVF	Category verbal fluency
10	DST	Digit symbol test
11	TMT A	Trail making test A
12	TMT B	Trail making test B
13	CDT	Clock drawing test
	PAL	Paired-associate learning of The Clinical Memory Test
14	PAL N1	The first learning trial of PAL
15	PAL N1- Simple part	Six simple word pairs of PAL N1
16	PAL N1- Difficult part	Six difficult word pairs of PAL N1
17	PAL-N2	The second learning trial of PAL
18	PAL N2- Simple part	Six simple word pairs of PAL N2
19	PAL N2- Difficult part	Six difficult word pairs of PAL N2
20	PAL-N3	The third learning trial of PAL
21	PAL N3- Simple part	Six simple word pairs of PAL N3
22	PAL N3- Difficult part	Six difficult word pairs of PAL N3
23	PAL-T	The total score of the three learning trials of PAL
	BDT	Block design test of the Aphasia Battery of Chinese
24	BDT N1	The first figure of BDT
25	BDT N2	The second figure of BDT
26	BDT N3	The third figure of BDT
27	BDT-T	The total score of BDT
28	Modified Luria TST	Modified Luria three-step task
	FC	Figure copying of the Aphasia Battery of Chinese
29	FC N1	The first figure of FC
30	FC N2	The second figure of FC
31	FC N3	The third figure of FC
32	FC N4	The fourth figure of FC
33	FC-T	The total score of four figures of FC
34	Gesture imitation	Imitation of seven hand gestures
35	Modified-Rey copy	Copy of a modified Rey-Osterrieth figure
36	Speech length	Sentence length of spontaneous speech
37	Speech time	Time of spontaneous speech

38	Semantic paraphasia	"Yes" or "No"
39	Phonemic paraphasia	"Yes" or "No"
40	Repetitive language	"Yes" or "No"
41	Word retrieval	Hesitation and delay in spoken production; "Yes" or "No"
42	Language output	"Fluent" or "nonfluent"
43	Language comprehension	Executing five commands
44	Repetition	Repeating three sentences
45	Object naming	The number of correctly named objects
46	Color naming	The number of correctly named colors
	AVLT	Auditory Verbal Learning Test--Huashan version
47	AVLT N1	The first learning trial of AVLT-H
48	AVLT N2	The second learning trial of AVLT-H
49	AVLT N3	The third learning trial AVLT-H
50	AVLT-L	Total score of three learning trials of AVLT-H
51	AVLT N4	The fourth short delayed free recall trial of AVLT-H
52	AVLT N5	The fifth long delayed free recall trial of AVLT-H
53	AVLT-T	Total score of AVLT-L, AVLT N4 and AVLT N5
54	AVLT N6	The sixth delayed category cue recall trial of AVLT-H
55	AVLT-RH	Recognitions hits of AVLT-H
56	AVLT-RF	Recognitions false of AVLT-H
	LMT	logical memory test of modified Wechsler Memory Scale
57	LMT N1	The first story of LMT
58	LMT N2	The second story of LMT
59	LMT N3	The third story of LMT
60	LMT-T	The total score of LMT
61	Modified-Rey recall	Modified Rey-Osterreith figure with a 10-minute free recall
62	Modified-Rey Recognition	Recognition of Modified Rey-Osterreith figure; "True" or "false"
63	Similarities	Similarities of the Wechsler Adult Intelligence Scale
64	Calculations	Calculations of the Wechsler Adult Intelligence Scale

Supplementary Table S2. Six neuropsychological subtests and they selected features.

**Number**	**Neuropsychological subtests**	**Selected features**
1	PAL	PAL N1<br>PAL N1- Simple part<br>PAL N1- Difficult part<br>PAL-N2<br>PAL N2- Simple part<br>PAL N2- Difficult part<br>PAL-N3<br>PAL N3- Simple part<br>PAL N3- Difficult part<br>PAL-T
2	AVLT-H	AVLT N1<br>AVLT N2<br>AVLT N3<br>AVLT-L<br>AVLT N4<br>AVLT N5<br>AVLT-T
3	LMT	LMT N2
4	Modified-Rey figure	Modified-Rey copy<br>Modified-Rey recall
5	DST	DST
6	TMT A	TMT A
